# Role of central and peripheral neuropeptides in escitalopram-induced weight gain and metabolic changes

**DOI:** 10.1192/j.eurpsy.2023.1040

**Published:** 2023-07-19

**Authors:** M. Y. Yilmaz

**Affiliations:** PSYCHIATRY CLINIC, VAN TRAINING AND RESEARCH HOSPITAL, VAN, Türkiye

## Abstract

**Introduction:**

Selective serotonin reuptake inhibitors (SSRI group antidepressant drugs) are not significantly different from tricyclic antidepressants and other antidepressants in terms of efficacy, but provide significant advantages in terms of side effects and toxicity. One of the most important side effects of antidepressant drugs is weight gain. However, there is not yet enough study on weight gain mechanism.

Nutrition and hunger-satiety circle are occured under the control of neuropeptids and hormones that are synthesized and secreted from the hypothalamic arcuate nucleus (ARC), adipose tissue and the pancreas.

In this study, we examined how escitalopram affects the body weight, the body mass index, the serum lipid profile, the liver function tests, the underlying molecular mechanisms of weight change, the relationships these mechanizms and the hypotalamic nutrition regulatory neuropeptides such as POMC, NPY, leptin, CCK and insülin that is a pancreatic hormone.

**Objectives:**

In order to understand the relationship between antidepressants and metabolic risk factors such as diabetes and obesity and to understand the underlying mechanisms, body weight, waist and hip circumference, POMC and NPY levels from hypothalamic nutrition regulating neuropeptides, CCK from peripheral neuropeptides, a pancreatic hormone insulin, and the effects of escitalopram use on these parameters were investigated.

**Methods:**

In this prospective study, 30 patients, who were decided to have escitalopram treatment and who met the inclusion criteria and continued the treatment for 12 weeks, were included in the study.

**Results:**

Weight, waist circumference increase and waist-hip ratio decreased significantly after 12 weeks. The decrease in neuropeptide level in POMC was significant.

**Conclusions:**

In our study, according to the insignificant change in lipid parameters it was thought that the use of escitalopram does not cause a metabolic change that would increase the risk in terms of metabolic syndrome and cardiovascular disease, despite the short study period. The decrease in POMC levels due to escitalopram use; It was thought that it may lead to weight gain by modulating eating behavior modulation.

**Disclosure of Interest:**

None Declared

